# Modeling Intraperitoneal Insulin Absorption in Patients with Type 1 Diabetes

**DOI:** 10.3390/metabo11090600

**Published:** 2021-09-03

**Authors:** Michele Schiavon, Claudio Cobelli, Chiara Dalla Man

**Affiliations:** 1 Department of Information Engineering, University of Padova, 35131 Padova, Italy; michele.schiavon@dei.unipd.it; 2 Department of Woman and Child’s Health, University of Padova, 35128 Padova, Italy; cobelli@dei.unipd.it

**Keywords:** mathematical modeling, parameter estimation, insulin delivery, intraperitoneal route, artificial pancreas

## Abstract

Standard insulin therapy to treat type 1 diabetes (T1D) consists of exogenous insulin administration through the subcutaneous (SC) tissue. Despite recent advances in insulin formulations, the SC route still suffers from delays and large inter/intra-subject variability that limiting optimal glucose control. Intraperitoneal (IP) insulin administration, despite its higher invasiveness, was shown to represent a valid alternative to the SC one. To date, no mathematical model describing the absorption and distribution of insulin after IP administration is available. Here, we aim to fill this gap by using data from eight patients with T1D, treated by implanted IP pump, studied in a hospitalized setting, with frequent measurements of plasma insulin and glucose concentration. A battery of models describing insulin kinetics after IP administration were tested. Model comparison and selection were performed based on model ability to predict the data, precision of parameters and parsimony criteria. The selected model assumed that the insulin absorption from the IP space was described by a linear, two-compartment model, coupled with a two-compartment model of whole-body insulin kinetics with hepatic insulin extraction controlled by hepatic insulin. Future developments include model incorporation into the UVa/Padova T1D Simulator for testing open- and closed-loop therapies with IP insulin administration.

## 1. Introduction

Type 1 diabetes (T1D) is an autoimmune disease characterized by the destruction of the insulin-secreting pancreatic beta cells, leading to chronic hyperglycemia. A 24/7 management of this disease is needed to keep blood glucose (BG) in the target range (70–180 mg/dL) and prevent diabetes-related long- and short-term complications [1].

The standard therapy of T1D consists of the subcutaneous (SC) administration of exogenous insulin, through a basal-bolus strategy: basal insulin is administered to keep patient glycemia within the normal range between meals and overnight, while boluses are injected before and after meals to prevent post-prandial hyperglycemic events. The SC injections are usually done using insulin pens (Multiple Daily Injection, MDI) or, more recently, insulin pump devices (Continuous Subcutaneous Insulin Infusion, CSII). These tools are then coupled with glucose sensing devices, such as Self-Monitoring Blood Glucose (SMBG) or, more recently, Continuous Glucose Monitoring (CGM) systems, helping patients to determine the amount of insulin to be administered to keep BG in a safe range. In addition, recently CSII and CGM devices have been used, in conjunction with a control algorithm implemented either on a tablet, a smartphone or directly into the pump, to develop a system able to automatically manage insulin dosing, the so-called Artificial Pancreas (AP) [2].

However, the non-physiologic nature of the SC insulin route led to suboptimal glucose control. In fact, while the adopted SC insulin delivery is convenient and minimally invasive, it is well-known that insulin appearance in plasma after SC administration is still suboptimal, mainly due to the delay and large inter-/intra-subject variability in its absorption [3] and the lack of a proper insulin-gradient across the liver.

To partially overcome these issues, the intraperitoneal (IP) route of administration can be used [4]. Two main approaches for IP insulin delivery have been developed: implantable pumps (Medtronic Minimed, Northridge, CA, USA) and the DiaPort system (Roche Diagnostics, Mannheim, Germany). The first one consists of a pump implanted in the abdomen, which delivers insulin via a catheter towards the liver; the second one consists of a flexible catheter placed in the peritoneal space with a small titanium port–body implanted into the SC tissue. Despite the higher invasiveness of IP vs. SC devices, these raised more interest in the diabetes technology community thanks to their ability to more closely mimic the physiological conditions occurring in healthy subjects. In fact, IP insulin delivery allows for the restoration of the portal-periphery insulin gradient and to avoid peripheral over-insulinization usually occurring with SC insulin delivery [5,6]. As a result, this allows for the improvement of glycemic control and, thus, patient satisfaction, compared to standard therapies [7,8,9,10]. Moreover, preliminary studies of closed-loop control using implanted IP insulin technology, both in silico as well as in vivo [11,12,13], have shown clinical feasibility and the potential to improve glucose control with respect to conventional SC insulin administration.

However, to the best of our knowledge, a mathematical model of IP insulin absorption and kinetics is still lacking. A better understanding of this process would be useful to help patients to properly adjust IP insulin therapy to improve glucose control. Moreover, a model of IP insulin absorption would be also an important component of in silico platforms, such as the UVa/Padova [14,15], the Cambridge [16] and the AIDA [17] T1D simulators, to develop and test new open- and closed-loop insulin treatment strategies. Here, we aim to develop a mathematical model of IP insulin absorption and kinetics using the data of patients with T1D treated by implanted IP pumps.

## 2. Materials and Methods

### 2.1. Database and Protocol

Eight subjects with T1D, treated by implanted IP pump infusing U-400 regular insulin (Insuplant; Sanofi-Aventis, Paris, France) and monitored by a subcutaneous continuous glucose monitoring (CGM) sensor, were studied in a hospitalized setting [13]. Participants’ characteristics were as follows (mean ± SD): 7M/1F, age 59.8 ± 8.7 years, BMI 26.4 ± 3.4 kg/m^2^, diabetes duration 31.7 ± 15.1 years, treatment duration by implanted pump 8.5 ± 7.4 years, A1C 6.8 ± 1.0%, and daily insulin requirement 0.60 ± 0.21 units/kg/day.

The study consisted of two randomized consecutive phases in random order: an open-loop (control) phase and a closed-loop phase. During the open-loop phase, patients were studied for 24 h (one day with 3 meals) with the IP insulin pump programmed based on patients’ standard basal-bolus insulin therapy. During the closed-loop phase, patients were studied for 48 h (2 days with 3 meals per day) with the IP insulin pump manually programmed by the patient to deliver 30% of meal insulin bolus approximatively 15 min before meal time, while the basal insulin infusion was automatically modulated by a Proportional–Integral–Derivative (PID) algorithm driven by the SC glucose sensor (CGM). In this work, only the open-loop phase data were used for model development and identification. No information about complications due to IP insulin administration was reported in the original paper, where one can find detailed protocol description [13].

### 2.2. Model Development

Modeling insulin kinetics after IP administration requires the development of a model of IP insulin absorption coupled with a model of whole-body insulin kinetics. In the following sections, a total of 9 models were proposed and tested. These were obtained by the combination of 3 different models of IP insulin absorption, coupled with 3 models of whole-body insulin kinetics which share the two-compartment structure [15] but differ in the description of hepatic insulin extraction (HE) [18]. In all models, insulin is assumed to pass from the IP space to the liver compartment.

#### 2.2.1. Model of IP Insulin Absorption

Three linear models of IP insulin absorption were tested. Model 1 was a single compartment absorption model:

(1) $$\{\begin{array}{ll} {\dot{Q}}_{ip1}\left(t\right)=-k_{a1}\cdot Q_{ip1}\left(t\right)+Inf\left(t\right) & Q_{ip1}\left(0\right)=Q_{ip1,0}\\ Ra_{I}\left(t\right)=k_{a1}\cdot Q_{ip,1}\left(t\right) & \end{array}$$

where *Q_ip_*_1_ (mU) is the insulin mass in the first IP compartment, *Inf* (mU/min) the insulin infusion rate from the IP pump. Parameter *k_a_*_1_ (min^−1^) represents the rate constant of insulin absorption from the IP space to the liver.

Model 2 was a two-compartment IP absorption model:

(2) $$\{\begin{array}{ll} {\dot{Q}}_{ip1}\left(t\right)=-k_{d}\cdot Q_{ip1}\left(t\right)+Inf\left(t\right) & Q_{ip1}\left(0\right)=Q_{ip1,0}\\ \begin{array}{l} {\dot{Q}}_{ip2}\left(t\right)=-k_{a2}\cdot Q_{ip2}\left(t\right)+k_{d}\cdot Q_{ip1}\left(t\right)\\ Ra_{I}\left(t\right)=k_{a2}\cdot Q_{ip2}\left(t\right) \end{array} & \begin{array}{l} Q_{ip2}\left(0\right)=Q_{ip2,0} \end{array} \end{array}$$

where *Q_ip_*_1_ and *Q_ip_*_2_ (mU) are the insulin masses in the first and second IP compartments, respectively. Parameter *k_d_* (min^−1^) represents the rate constant of insulin distribution between the two IP compartments and *k_a_*_2_ (min^−1^) the rate constant of insulin absorption from the second IP compartment to the liver.

Finally, Model 3 is, again, a two-compartment model where, at variance with Model 2, insulin is assumed to be absorbed both from the first IP compartment, through the rate constant *k_a_*_1_ (min^−1^), as well as from the second IP compartment, through the rate constant *k_a_*_2_ (min^−1^):

(3) $$\{\begin{array}{ll} {\dot{Q}}_{ip1}\left(t\right)=-\left(k_{a2}+k_{a1}\right)\cdot Q_{ip1}\left(t\right)+Inf\left(t\right) & Q_{ip1}\left(0\right)=Q_{ip1,0}\\ \begin{array}{l} {\dot{Q}}_{ip2}\left(t\right)=-k_{a2}\cdot Q_{ip2}\left(t\right)+k_{a2}\cdot Q_{ip1}\left(t\right)\\ Ra_{I}\left(t\right)=k_{a1}\cdot Q_{ip1}\left(t\right)+k_{a2}\cdot Q_{ip2}\left(t\right) \end{array} & \begin{array}{l} Q_{ip2}\left(0\right)=Q_{ip2,0} \end{array} \end{array}$$

In all the models, the rate of insulin absorption from the IP to the liver compartment was represented by *Ra_I_* (mU/min).

#### 2.2.2. Model of Whole-Body Insulin Kinetics

The proposed models of IP insulin absorption were coupled with the two-compartment model of whole-body insulin kinetics included in the UVA/Padova T1D simulator [15]:

(4) $$\{\begin{array}{ll} {\dot{Q}}_{p}\left(t\right)=-\left(m_{2}+m_{4}\right)\cdot Q_{p}\left(t\right)+m_{1}\cdot Q_{l}\left(t\right) & Q_{p}\left(0\right)=Q_{pb}\\ \begin{array}{l} {\dot{Q}}_{l}\left(t\right)=-\left(m_{1}+m_{3}\left(t\right)\right)\cdot Q_{l}\left(t\right)+m_{2}\cdot Q_{p}\left(t\right)+Ra_{I}\left(t\right)\\ I_{p}\left(t\right)=\frac{Q_{p}\left(t\right)}{V_{I}} \end{array} & \begin{array}{l} Q_{l}\left(0\right)=Q_{lb} \end{array} \end{array}$$

where *Q_p_* and *Q*_l_ (mU) are the insulin masses in plasma and liver compartment, respectively, and *I_p_* (mU/L) is the plasma insulin concentration. As already anticipated, at variance with the peripherally administered insulin, which is usually assumed to be absorbed into the plasma compartment, here IP administration is assumed to appear in the liver compartment, which is close to the anatomical site of the IP plant. Parameter *V_I_* (L) represents the volume of insulin distribution, *m*_2_ (min^−1^) the fractional rate of hepatic plasma flow [17], *m*_1_ the rate of insulin distribution from liver to plasma, *m*_4_ (min^−1^) the rate of insulin degradation in the periphery and *m*_3_ (min^−1^) the rate of insulin degradation in the liver which is given by:

(5) $$m_{3}\left(t\right)=\frac{HE\left(t\right)}{1-HE\left(t\right)}\cdot m_{1}$$

where *HE* represents the hepatic extraction of insulin. From a combination of model parameters, the post-hepatic insulin clearance CL (L/min), i.e., the clearance of insulin peripherally administered, can also be calculated as:

(6) $$CL=\left(HE_{b}\cdot m_{2}+m_{4}\right)\cdot V_{I}$$

where *HE_b_* is the basal hepatic insulin extraction (the subscript *b* denotes the basal state).

Three models of *HE* were tested in this work: in Model A, *HE*(*t*) is assumed to be constant (and equal to *HE_b_*) throughout the experiment as in [15]; in Model B, *HE*(*t*) is regulated by glucose concentration in plasma, as proposed in [18]:

(7) $$HE\left(t\right)=-a_{G}\cdot \left(G\left(t\right)-G_{b}\right)+HE_{b}$$

where *a_G_* (dL/mg) represents the control of glucose on *HE*(*t*) and *G*(*t*) the plasma glucose concentration (mg/dL); in Model C, *HE*(*t*) is regulated by insulin mass in the liver compartment:

(8) $$HE\left(t\right)=-a_{I}\cdot \left(Q_{l}\left(t\right)-Q_{l,b}\right)+HE_{b}$$

where *a_I_* (mU^−1^) represents the control of hepatic insulin.

### 2.3. Model Identification

The a priori identifiability of all the models was assessed using the software DAISY [19]. All models resulted a priori identifiable once m_2_ is assumed to be known (fixed to 0.268 min^−1^ [20]). Moreover, in Model 2, without loss of generality, we assumed *k_d_* ≥ *k_a_*_2_ since these two parameters are interchangeable (identifiable but not uniquely).

All the models were numerically identified on plasma insulin concentration data using a Bayesian Maximum a Posteriori (MAP) estimator [21]. Specifically, for all the models, *a priori* information on whole-body insulin kinetics parameters *V_I_*, *m*_1_, *CL* and *HE_b_* has been used to improve their numerical identifiability [15]. In addition, to assess intra-subject (between-meals) variability in the IP insulin absorption, parameters of the IP absorption model were allowed to vary meal-by-meal (breakfast, lunch and dinner). Measurement error on plasma insulin data was assumed to be uncorrelated, Gaussian, with zero mean and known standard deviation, as reported in [22].

Of note, on some occasions, models can be simplified when one of the following conditions occurs: (i) in Model 2, *k_a_*_2_ = *k_d_* when the two parameters are virtually identical, i.e., the absolute relative difference was less than 1%; (ii) in Model 3, *k_a_*_1_ = 0 when *k_a_*_1_ was less than 10^−3^ min^−1^.

Model identification and statistical analysis were performed in Matlab^®^ (R2016a) and the ode45 solver, implemented in Matlab^®^, was used to integrate the model differential equations [23].

### 2.4. Model Assessment and Comparison

Model ability to predict the data was assessed by checking the randomness of weighted residuals, using Runs test. The numerical (a posteriori) identifiability of model parameters [21] was assessed by calculating the coefficient of variation (CV, %) of estimated parameters: poor precision of estimated parameter is defined when its CV is above 100%. If all the previous criteria were satisfied, model comparison was performed according to the Bayesian Information Criterion (BIC) [24]. Of note, the physiological plausibility of model parameters was not used as a criterion since all model parameters were physiologically plausible.

### 2.5. Statistical Analysis

Model parameters are presented as median [25th, 75th] percentiles. Paired two-samples comparison was done by Wilcoxon Signed-Rank test and Kruskal–Wallis test was used for multiple comparison. Significance level was set at *p* = 0.05 for all the statistical tests.

## 3. Results

### 3.1. Model Comparison

As shown in Table 1, all models of IP insulin absorption provide acceptable/good residual independence, regardless from the specific description of hepatic extraction adopted in the whole-body insulin kinetics model, with a number of subjects passing the Runs test ranging from 6/8 to 8/8. In particular, Model 3A, i.e., given by the combination of Model 3 of IP insulin absorption and Model A of whole-body insulin kinetics, was not able to achieve residual independence in 2 out of 8 subjects; Models 1A, 2A and 2C were not able to achieve residual independence for one subject; while all the subjects passed the Runs test with Models 1B, 2B, 3B, 1C and 3C.

Among the models achieving the best results in residual independence, Model 1C and 3C provided the highest number of model parameters estimated with precision (coefficient of variation, CV, below 100% in 97% and 96% of the cases, respectively), while Model 1B, 3B and 2B provided precise estimates in only 92%, 89% and 80% of the parameters, respectively. In particular, the poor precision of the estimated model parameters achieved with Models B was mainly due to the inability of estimating the glucose contribution on hepatic insulin extraction (*a_G_*), which was badly estimated (CV >> 100%) in five out of eight subjects for both Models 1–3.

Finally, between Model 1C and 3C, the lowest value of the Bayesian Information Criterion (BIC) index, i.e., the ability to best predict the experimental data with the minimum number of parameters, was achieved by Model 3C.

Summing up, among the models achieving the best results in terms of residual independence and precise parameter estimates, the combination of IP Model 3 and the whole-body insulin kinetics Model C resulted as the most parsimonious one (lowest BIC).

**Figure 1:**
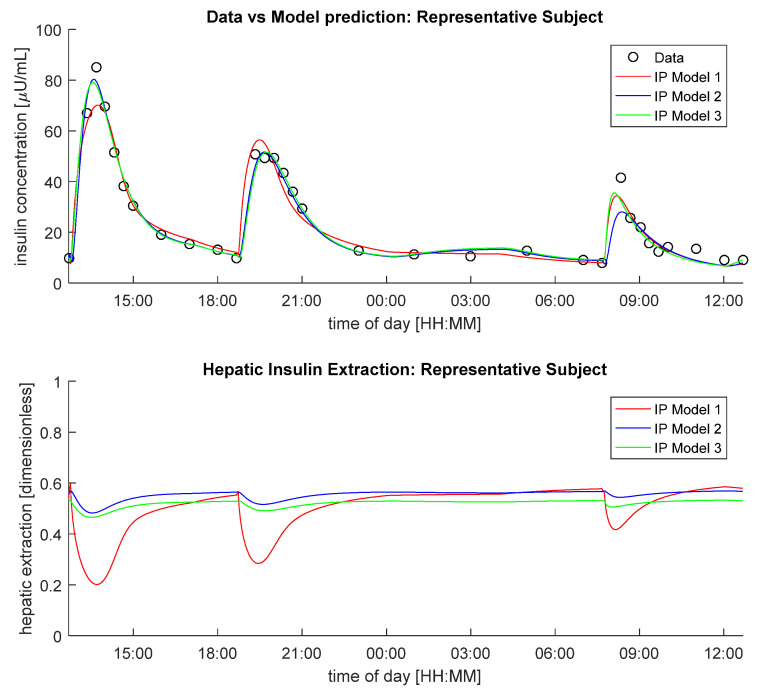
Insulin concentration data in a representative subject (black circles) vs. model predictions (continuous lines, top panel) and predicted hepatic insulin extraction (bottom panel) provided by IP Model 1(red line), IP Model 2 (blue line) and IP Model 3 (green line), coupled with Model C.

In Figure 1, data vs. model predictions of IP Models 1–3, assuming hepatic insulin extraction controlled by hepatic insulin (Model C), is reported (top panel), together with the predicted hepatic insulin extractions (bottom panel) in a representative subject. Of note, despite intra-subject (between-meals) variability of IP model parameters was accounted in each model, only Model 3 was able to predict all plasma insulin concentration excursions well.

### 3.2. Performance of the Selected Model

**Figure 2:**
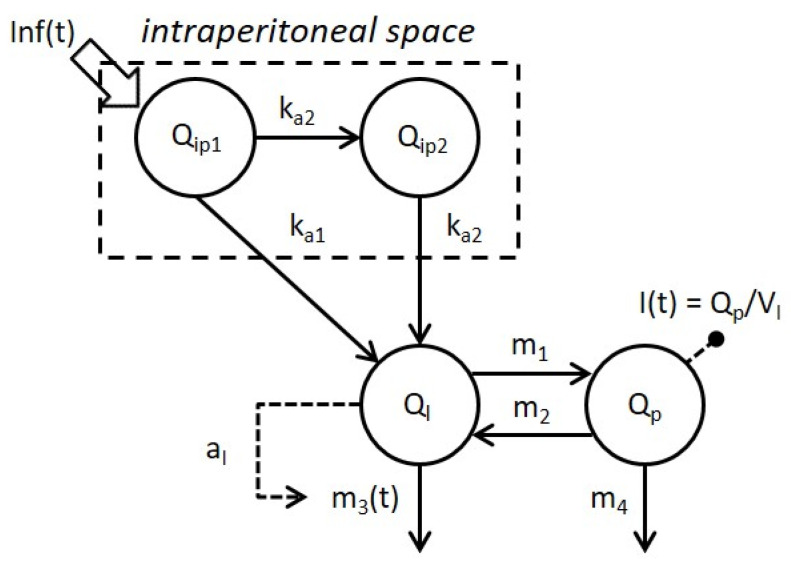
Schematic representation of the selected model combining Model 3 of intraperitoneal absorption of insulin and Model C of whole-body insulin kinetics. Model compartments are represented by white circles, fluxes and control signals are indicated by continuous and dashed arrows respectively. Signals Inf(t) and I(t) represent the intraperitoneal insulin administration and plasma insulin concentration, respectively.

The model selected (3C) is reported in Figure 2.

**Figure 3:**
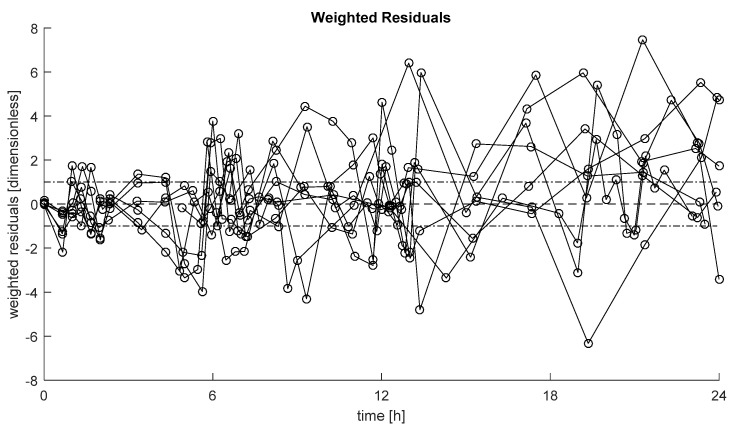
Weighted residuals of the selected model in each subject. Dashed line represents zero value, while dashed-dotted lines represent [−1, +1] interval.

Weighted residuals, defined as (data-model prediction)/SD, with SD standard deviation of the measurement error on insulin data [22], of the selected model in each subject are reported in Figure 3. These show the ability of the model to describe the data in most of the cases, with no long sequences of samples above or below zero (randomness of residuals achieved in 100% of the subjects).

The median [25th, 75th] percentiles (median CV) of estimated model parameters are reported. The constant rate of absorption from the first IP compartment *k_a_*_1_ = 0.018 [0.009, 0.029] min^−1^ (18%), 0.004 [0.002, 0.010] min^−1^ (39%) and 0.012 [0.004, 0.027] min^−1^ (27%), at breakfast, lunch and dinner, respectively; while from the second IP compartment *k_a_*_2_ = 0.028 [0.021, 0.037] min^−1^ (19%), 0.028 [0.019, 0.030] min^−1^ (13%) and 0.024 [0.020, 0.032] min^−1^ (20%), at breakfast, lunch and dinner, respectively. The volume of insulin distribution *V_I_* = 3.4 [3.3, 3.6] L (17%), the constant rate of insulin distribution from liver to plasma *m*_1_ = 0.15 [0.13, 0.20] min^−1^ (43%), the post-hepatic insulin clearance *CL* = 1.16 [0.93, 1.29] L/min (32%), the basal hepatic insulin extraction *HE_b_* = 0.59 [0.56, 0.64] (9%), and the control of hepatic insulin on its own extraction *a_I_* = 16 × 10^−5^ [6 × 10^−5^, 29 × 10^−5^] mU^−1^ (49%). However, no statistically significant difference was detected between meals in *k_a_*_1_ and *k_a_*_2_ using the Kruskal–Wallis test. Of note, in 23% of the cases, parameter *k_a_*_1_ collapsed to zero (estimated below 10^−3^ min^−1^).

## 4. Discussion

IP insulin delivery recently raised interest in the diabetes community as a promising alternative to the conventional SC route [11,13] thanks to its faster pharmacokinetics and pharmacodynamics [5,6]. These have become particularly important, since in the last decade it has become clear that SC insulin administration is, to date, the bottleneck for the development of a safe and effective automatic glucose control. Another important take-home message of the last decade was that an effective design of an artificial pancreas algorithm must pass through in silico testing before being used in in vivo trails. However, to the best of our knowledge, a model describing the IP insulin absorption and kinetics is still not available in the literature. Here, we aim to fill this gap exploiting data from a population of eight subjects with T1D treated with an implanted IP insulin pump in a hospitalized setting [13]. A battery of models of both IP insulin absorption and whole-body insulin kinetics, including different descriptions of hepatic insulin extraction, were proposed and tested. The model selected as the best was made by a linear two-compartment model, describing the absorption from both the first and the second IP compartment (Model 3), coupled with a two-compartment model of insulin kinetics, similar to that included in the UVa/Padova T1D simulator [15], but with hepatic insulin extraction controlled by insulin in the liver (Model C). The model well predicted the data in most of the subjects (Figure 3) with model parameters estimated with precision (Table 1).

Model parameters of IP insulin absorption (*k_a_*_1_ and *k_a_*_2_) were allowed to vary among meals but, despite this, these were not significantly different among meals, as detected by Kruskal–Wallis test. In particular, the overall, i.e., from all meals, estimated model parameters were: *k_a_*_1_ = 0.010 [0.004, 0.024] min^−1^ (estimated in 77% of the subjects) and *k_a_*_2_ = 0.028 [0.017, 0.033] min^−1^. Regarding the hepatic insulin extraction, at variance with the model reported in [18], here a control by glucose concentration on HE was detectable in only 3 out of 8 subjects. In particular, we found that the percentage hepatic insulin extraction decreased when insulin levels in the liver increased. This suggests that the hepatic insulin extraction process saturates at high intrahepatic insulin level, in agreement with what reported in previous studies [25].

**Figure 4:**
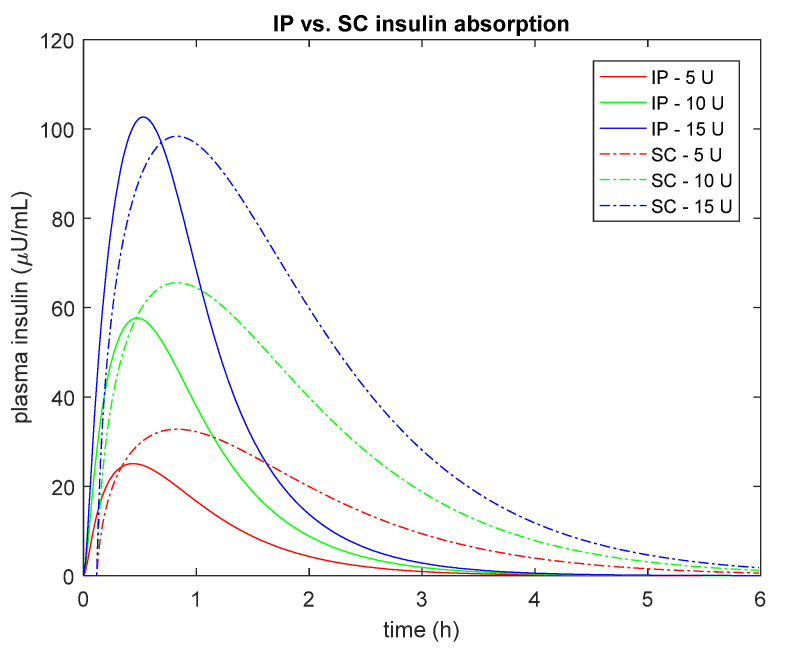
Simulated plasma insulin concentration after different bolus injection amounts (5U, red; 10U green and 15U blue) of U-400 regular insulin using the proposed IP model (continuous line) vs. U-100 fast-acting insulin concentration obtained with the SC model incorporated into the UVa/Padova T1D Simulator [15] (dashed line). Median estimated parameters were used to simulate both the IP and SC routes.

Overall, these results are in line with the expected faster IP absorption than the conventional SC route also when using fast-acting insulin analogues [3]. In fact, if one simulates plasma insulin concentration after a bolus injection of U-400 regular insulin using the proposed IP model vs. the same amount of insulin after a bolus of U-100 fast-acting insulin using the SC model incorporated into the UVa/Padova T1D Simulator [15], one can observe a faster appearance/disappearance of plasma insulin after IP vs. SC administration (Figure 4). The nonlinear behavior of the IP model is also evident from Figure 4, where the peak of plasma insulin concentration was lower with the IP than SC absorption for both a 5 and 10 U boluses, while it is higher with the IP than SC for the 15 U bolus.

The first major limitation of this work is the small number of subjects available in the database. Hence, further work is needed to validate or falsify the model on a larger dataset. A second limitation of this work is that, despite the model accounting for possible intra-subject (between-meals) variability in IP insulin absorption, in a few cases, it was not able to perfectly predict plasma insulin data (Figure 3). This could be due to unaccounted delays in IP insulin appearance after IP administration, similarly to what observed after SC injection [3]; possible nonlinearities in IP absorption such as those due to insulin volume; unaccounted intra-day variability of whole-body insulin kinetic parameters, etc. Another possible limitation is that, in this work, the whole-body insulin kinetics were described by a two-compartment model, even if a more physiological but complicated three-compartment model has also been proposed [20]. The choice was driven by the necessity to keep such a model as simple as possible, to favor both the a priori and the a posteriori identifiability, but also to make the model easy to incorporate in the UVa/Padova T1D Simulator, as done in [26,27]. This is particularly important to enable the in silico testing of a future closed-loop control algorithm for next generation SC-IP artificial pancreas. Finally, in this work U-400 regular insulin was adopted as usual formulation employed for implanted insulin pumps, allowing for the reduction in injected insulin volumes and less frequent refills of the reservoir. Hence, the model needs to be further tested if different insulin formulations are used.

Future work will assess the IP absorption model in combination with other models of whole-body insulin kinetics. Finally, future developments of the present work will also include testing the validity of the model to describe IP insulin absorption using other IP insulin administration systems, such as the DiaPort by Roche (Second Generation, Roche Diagnostics, Mannheim, Germany), as well as more recent devices [28].

## 5. Conclusions

In this work, a battery of models of IP insulin absorption and kinetics were proposed and tested on subjects with T1D, treated by implanted IP pump. The selected model (Figure 2) is a linear two-compartment model for IP absorption, with absorption from both IP compartments, coupled with a two-compartment model for insulin kinetics assuming hepatic insulin to control its own extraction. Overall, the median constant rate of absorption from the first (*k_a_*_1_) and second (*k_a_*_2_) IP compartments were 0.010 min^−1^ (estimated in 77% of the subjects) and 0.028 min^−1^, respectively; and a saturation of hepatic insulin extraction governed by intrahepatic insulin levels was suggested. The model represents the best trade-off between the ability to predict the data and the minimum number of precisely estimated parameters, so that 96% of model parameters were estimated with a coefficient of variation less than 100%. Future work will include testing the model on larger datasets and incorporating it into the UVa/Padova T1D Simulator for testing open- and closed-loop therapies with IP insulin administration.

## Figures and Tables

**Table 1:** Summary results of model comparison.

Intraperitoneal Absorption Model	Whole-Body Kinetic Model	Residual Independence (*)	Parameters Estimated with Coefficient of Variation (CV) < 100%	BIC (**)
1	A	7/8	100%	183
2		7/8	86%	178
3		6/8	97%	177
1	B	8/8	92%	168
2		8/8	80%	176
3		8/8	89%	177
1	C	8/8	97%	171
2		7/8	91%	176
3		8/8	96%	170

* Number of subjects out of the total. ** Bayesian Information Criterion (BIC): median values.

## Data Availability

Restrictions apply to the availability of these data. Data were obtained from Eric Renard and are available from the authors with the permission of Eric Renard.
